# Dietary pattern and its association with iodine deficiency among school children in southwest Ethiopia; A cross-sectional study

**DOI:** 10.1371/journal.pone.0221106

**Published:** 2019-08-13

**Authors:** Hamid Yimam Hassen, Melkamu Beyene, Jemal Haider Ali

**Affiliations:** 1 Department of Public Health, Mizan Tepi University, Mizan Teferi, Ethiopia; 2 Department of Nutrition, School of public health, Addis Ababa University, Addis Ababa, Ethiopia; University of Ghana, GHANA

## Abstract

**Background:**

Despite the universal iodization of salt in Ethiopia, iodine deficiency disorder remains a major public health problem and continued to affect a large segment of the population. It is thus essential to assess factors contributing to the unacceptably high endemic goiter rate in the country and avail evidence for further additional interventions. In line with this, we examined the association of dietary pattern and iodine deficiency among school-age children in Ethiopia.

**Method:**

We conducted a school-based cross-sectional study among 767 children aged 6 to 12 in southwest Ethiopia. We collected socio-demographic and other important health related information using a pre-tested structured questionnaire through the interview. Dietary pattern of children was measured using modified Hellen Keller’s food frequency questionnaire. We measured iodine deficiency using urinary iodine concentration level and total goiter rate, according to the World Health Organization threshold criteria. We used a multivariate linear regression model to identify dietary and sociodemographic factors that affect urinary iodine level among children.

**Result:**

Out of the 767 children included in the study, 12% and 4% of children have grade 1 and grade 2 goiter respectively, making the total goiter rate 16%. While the prevalence of iodine deficiency based on urinary iodine concentration is 58.8% of which 13.7% had severe, 18.6% had moderate and 26.5% had mild form. The proportion of children who consumed godere/taro root/, banana, corn, Abyssinian cabbage, and potato, respectively at daily basis 57.8%, 53.1%, 37.9%, and 31.2%, respectively. Age (β = -0.7, 95%CI = -1.1, -0.4), sex (β = -22.3, 95%CI = -33.8, -10.8), consumption of taro root (β = -27.4, 95%CI = -22.9, -31.8), cabbage (β = -11.7, 95%CI = -5.7, -17.6), Abyssinian cabbage (β = 12.4, 95%CI = 6.7, 18.2), and banana (β = 5.6, 95%CI = 0.01, 11.2) significantly associated with urinary iodine level.

**Conclusion:**

Iodine deficiency remains an important public health problem in southwest Ethiopia. Over-consumption of goitrogenic foods and under-consumption of iodine-rich foods were prevalent and associated with lower urinary iodine level. Therefore, dietary counseling apart from universal salt iodization is recommended.

## Introduction

Iodine is an essential micronutrient that is needed for the production of thyroid hormone. Thyroid hormone is needed to support various physiological functions for normal growth and is particularly critical for cognitive and neurodevelopment during fetal life, infancy, and childhood [1–4]. Nevertheless, its deficiency is associated with a wider range of health problems in all age groups [3, 5]. Iodine deficiency (ID) occurs when iodine intake falls below the recommended level and the thyroid gland is no longer able to synthesize sufficient amounts of thyroid hormone. It is among the most common preventable cause of mental retardation in children [6, 7] and could result in decreased physical growth, poor school performance and lower body resistance to infections [8–11].

Iodine deficiency is a global public health problem which is endemic in Europe, Asia, South and Central America, and Eastern Africa specifically among highland regions [12, 13]. Pregnant mothers and school children are among disproportionately vulnerable groups of iodine deficiency [14]. According to the World Health Organization (WHO) estimates, approximately 37% of school-age children worldwide are at risk of insufficient iodine intake [3]. Furthermore, in 2016 ID was responsible for 22,00 deaths, and caused 25% of the Disability Adjusted Life Years (DALY’s) occurring in Africa [3, 15] indicating to affect significantly the socioeconomic development of the country at large [14].

In Ethiopia, iodine deficiency disorder remains a major public health problem which continues to affect a large segment of the country’s population. The prevalence greatly varies across different regions in the country, and some areas are reported to have a total goiter rate of 71% [16].

Iodine deficiency is mainly caused by either low iodine content in the diet or problem of absorption in the human body [17]. The richest dietary sources of iodine are seafood, seaweed and iodized salt [18], and foods of animal origin, including meat and milk can also constitute a significant source of iodine if animals have grazed on iodine sufficient soils [19]. Although it is primarily caused by insufficient dietary intake, other food items referred to as goitrogens like cabbage, kale, cassava, millet, godere/taro root (*Colocasia Esculenta*) have been suggested to interfere with the proper functioning of thyroid hormone synthesis and utilization [20–23]. Furthermore, the source of water, co-existing micronutrient deficiencies, poor socioeconomic status, low maternal education, and poor hygienic practices are also associated with iodine deficiency [24–26]. Moreover, studies showed that gradual and higher heat exposure could lead to iodine loss from the salt fortified with iodine [27, 28].

Although the Ethiopian Ministry of Health designed a micronutrient guideline and endorsed a proclamation for ensuring the availability of iodized salt, the prevalence of iodine deficiency is still a significant public health problem. It is thus apparent that a study is needed to examine the factors contributing to the unacceptably high endemic goiter rate in the country and avail evidence for further additional interventions among school-aged children in Ethiopia. Therefore, this study aimed to identify the dietary pattern, type, and frequency of food items that determine iodine deficiency among school children in southwest Ethiopia.

## Methods and materials

### Study setting

We conducted a school-based study among children aged 6 to 12 years in primary schools in Bench-Maji zone, Southwest Ethiopia. The main food types consumed in the study area are maize, taro root, fruits such as banana, mango, avocado, Abyssinian cabbage, and enset. Sorghum, teff, wheat, and barley are also cultivated to a significant extent. While cattle, shoats, and poultry are produced in limited numbers. Cash crops include coffee, fruits, spices such as coriander and ginger, and honey. The zone is located at a latitude of 6.45994, longitude 35.30549 and 747 to 2494 meters height above sea level.

### Study design and sampling procedures

The present study employed a school-based cross-sectional study design among children aged 6 to 12 years in southwest Ethiopia. The sample size was determined using a single proportion formula based on the estimation of the proportion of iodine deficiency in southeast Ethiopia, 57% [29], absolute precision of 5.0 and 95% level of confidence, a design effect of 2.0 and a non-response rate of 5% yielded a total of 792 subjects. A multi-stage sampling technique was used for the selection of study participants. In the first stage, three districts were selected randomly among the available ten districts in the Bench-Maji zone. Subsequently, four primary schools were selected from each district using simple random sampling. A total of 4482 students within the age group of 6 to 12 years were available from 12 primary schools. Later, 792 school age children was selected from 12 primary schools using proportionate to population size sampling. In collaboration with schools teacher-parent association, mothers/caregivers of the selected children were invited to participate in the study after a detailed explanation of the study.

### Exposure and Covariate measurement

We collected socio-demographic information of the mother/caregiver, including age, sex, ethnicity, educational attainment, and place of residence using an interviewer-administered questionnaire. The age of the child was transcribed from the verbal report of the mother/caregiver. For children with an unknown day of birth, the 15th day of the month was used. To compute the household wealth index, all the asset that the family owned was recorded using a structured questionnaire. The type and frequency of food a child eats were recorded using modified Hellen Keller’s food frequency questionnaire that was previously used in Ethiopia [30]. Salt samples were collected from the house of the sampled children and the test was performed using a rapid spot field test kit and values were compared with the color chart.

### Outcome measurement and biochemical analysis

Iodine deficiency manifested as goiter was examined and graded by palpation with the help of trained public health officers using standard procedures as per the criteria of WHO/UNICEF/ICCIDD [3]. Accordingly, goiter was graded as; *grade 0***:** no palpable or visible goiter; *grade 1***:** a goiter that is palpable, but not visible when the neck is in the normal position and *grade 2***:** swelling in the neck, which is visible when the neck is in a normal position.

To measure the urinary iodine concentration (UIC), about 5 ml of spot urine samples were collected from all children in a properly labeled and sterile screw-capped plastic vials. These vials immediately transferred to the thermos-cool box containing ice bags and transported to the Ethiopian Public Health Institute (EPHI) Laboratory which is found in Addis Ababa for biochemical analysis. The samples were kept at 4°C in a refrigerator with all precautionary measures until analysis performed.

Analysis of urinary iodine was performed using the spectrophotometric procedure, based on ammonium persulfate method which was suggested and approved by WHO/UNICEF/ICCIDD [3]. This involves the spectrophotometric analysis (measuring the absorbance) of a reaction medium, which utilizes iodine as a catalyst. During this reaction, cerric ammonium sulfate (yellow in color), one of the reactants is reduced to cerrous (colorless) form, which uses iodine as a catalyst. The absorbance value/ optical density (OD) at 405 nm gives a clue about the iodine content of the urine sample. It indicates that the more the absorbance value is the lesser the iodine content. The absorbance values and iodine concentration in the urine have an inverse relationship. The absorbance value can be used for the determination of the actual iodine concentration using a standard graph prepared by using a range of standard KIO_3_ solutions.

Among several methods of assessment, urinary iodine is considered as a clear cut indicator of iodine nutritional status. WHO/UNICEF/ICCID have made some guidelines regarding categorizing individuals to different iodine nutritional groups based on urinary iodine level [3]. Accordingly, <20 μg iodine/l of urine indicates severe iodine deficiency, 20–49.9 μg iodine/l indicates moderate iodine deficiency, 50–99.9 μg iodine/l indicates mild iodine deficiency, 100–199.9 μg iodine/l indicates an adequate or sufficient level, 200–300 μg iodine/l indicates above the requirement and >300 μg iodine/l indicates an excessive level of iodine in the body [31].

### Data collection and quality management

We recruited six data collectors, who are public health professionals and working as a clinician and two supervisors who had experience in data collection and supervision. We conducted a two-day comprehensive training on the administration of the questionnaire, interview skills, thyroid examination, urinary samples taking and temporary storage. Two authors (H.Y.H and M.B.) translated the questionnaire into the Amharic language (local language) and reviewed it together with data collectors who were residents in the study area. We also conducted a pre-test in an adjacent zone called *Sheka*, which has similar socioeconomic, cultural, and geographical characteristics as those in the study area. To avoid the existence of misperception of the respondents for the questions, we assessed validity of the data collection tool during pilot test in neighboring zones. Besides, we also performed a reliability test using the data from the pilot test and the result showed acceptable reliability (reliability coefficient (alpha) = 0.87). The collected data were checked by the investigator on a daily basis for any incompleteness and/or consistency and timely action was made.

### Data processing and analysis

The collected data were checked for its completeness, coded and entered using EpiInfo version 7, and analysis was done using R statistical package version 3.5.1. Descriptive statistics including mean, median and frequencies were carried. We computed wealth index using principal component analysis (PCA) of the reported ownership of household assets and proxy indicators of living standard variables. Later, using quintiles, we categorized the wealth status into three groups (poor/low-40%, average/middle-40%, and rich/upper-20%). The chi-square and Mann-Whitney U test was used to test the association of sex with the magnitude of goiter and urinary iodine level respectively. While a Kruskal-Wallis test was performed to examine the association of age categories and wealth index with urinary iodine level. Bivariate analysis was conducted to identify the association of various factors with iodine deficiency and to identify variables for multivariate analysis. Finally, a multivariate linear regression analysis was applied to identify independent factors associated with iodine deficiency. As children selected from several schools, individual data were likely to be clustered within the 12 different schools, which could affect the association of the predictor variables with the outcome. We accounted for such possible non-random differences within schools (clusters) using multilevel logistic regression techniques. However, the multilevel analysis identified nearly the same intercept, coefficient, and confidence intervals as the standard multivariable linear regression analysis. Moreover, the variability test also showed there is no significant variation between schools (intra-class correlation (ICC) ~ 0). Hence we presented the results of the standard linear regression model. Coefficients (β) with 95% CI was used to present the strength and significance of the association.

### Participant consent and ethical approval

The protocol of this study obtained ethical clearance from the ethical review committee of Mizan-Tepi University. Subsequently the Benchi-Maji zonal health office and the three district health administrations (Mizan-Aman Health Office, Debrework district Health Office and Sheko district Health Office) also approved the study and gave support letters. Data were collected from all children after informed written parental consent and child assent was obtained at their residence after the nature and aim of the study was explained to them in their local language. All children who were iodine deficient were linked to the closest health centers.

## Result

Out of the 792 mother-children pairs recruited, 767 were volunteered and interviewed that made the response rate of 96.8%.

### Socio demographic characteristics of the mother/caregiver

The mean age and standard deviation of the mothers/caretakers is 39.5(±4.2) years. The majority (89.4%) are married and 44.2% of them are from Bench ethnic groups. About two-thirds (65.4%) are from urban settings with only slightly above half (51.8%) have formal education. Less than half (42.2%) are farmers and nearly half (45.8%) earn a monthly income of less than 1000 ETB. Nearly two-thirds (65.4%) have a family size of five and above. (Table 1).

**Table 1:** Socio-demographic characteristics of mothers/caretakers, southwest Ethiopia, 2016.

Socio-demographic characteristic	Level	Frequency	Percent
**Marital status**	Married	686	89.4
	Single	28	3.6
	Divorced	22	2.9
	Widowed	23	3.0
	Separated	8	1.0
**Religion**	Protestant	471	61.4
	Orthodox	204	26.6
	Muslim	87	11.3
	Other^a^	5	0.6
**Ethnicity**	Bench	339	44.2
	Amhara	158	20.6
	Sheko	96	12.5
	Keffa	101	13.2
	Tigre	20	2.6
	Other^b^	53	6.9
**Residence**	Urban	502	65.4
	Rural	265	34.6
**Educational status**	No read and write	324	42.2
	Read and write (non-formal)	46	6.0
	Primary (grade 1–8)	295	38.5
	Secondary (grade 9–12)	70	9.1
	12 and above	32	4.2
**Occupation**	Housewife	195	25.4
	Farmer	324	42.2
	Merchant	123	16.0
	Gov’t employee	62	8.1
	Private employee	8	1.0
	Private own business	35	4.6
	Laborer	12	1.6
	Other^c^	8	1.0
**Household monthly income (in ETB)**	Less than 1000	351	45.8
	1000–2000	212	27.6
	2001–3000	133	17.3
	3001–4000	37	4.8
	4000 and above	34	4.4
**Family size**	3 and less	81	10.6
	4	184	24.0
	5	215	28.0
	6	139	18.1
	7 and above	148	19.3
**Total**		**767**	**100.0%**

^a^- Catholic, Adventist, atheist,

^b^- Oromo, Silte, Gurage,

^c^- Carpenter, tailor

ETB = Ethiopian Birr which is equivalent to 0.037USD

### Iodized salt Awareness and source of water

Half (50.8%) of respondents have ever heard about iodized salt, of which 209 (53.6%) mentioned their sources of information are health workers. Over half (59.5%) are able to differentiate between iodized and ordinary salt from the label of the package. Almost all (94.5%) of households use salt during food preparation and over half (54.1%) add salt to the food at the end of cooking. More than half (56.4%) of the households use spring water as the main source of drinking and cooking. (Table 2).

**Table 2:** Awareness of mothers/caretakers about iodized salt and water source, southwest Ethiopia, 2016.

Awareness about iodized salt	Response categories	Frequency	Percent
**Ever heard about iodized salt**	Yes	390	50.8
	No	377	49.2
**Source of information (N = 390)**	Health workers	209	53.6
	Friends	23	5.9
	Relative	6	1.5
	Family	36	9.2
	Advertisements	111	2.8
	Others	5	1.3
**Able to differentiate iodized salt (N = 390)**	Don’t know	115	29.5
	See the labeling	232	59.5
	By the taste	1	0.3
	By color	1	0.3
	Others	41	10.5
**Use salt for food**	Yes	725	94.5
	No	42	5.5
**Time added salt to the food (N = 725)**	Initial	15	2.1
	Mid-point	153	21.1
	At the end	392	54.1
	After completed	165	22.8
**Source of water for drinking**	Pipe	294	38.3
	Spring	433	56.4
	Well	22	2.9
	River	7	0.9
	Other	11	1.4

### Dietary intake of children

The feeding pattern of the children was also assessed using the modified Hellen Keller food frequency questionnaire. Accordingly, frequent consumption of Abyssinian cabbage, taro root, banana, corn, and potato were observed. The proportion of children who consumed taro root, banana, corn, Abyssinian cabbage, and potato on daily basis was 47.8%, 57.8%, 53.1%, 37.9%, and 31.2%, respectively, indicating higher consumption of root and vegetables. While the proportion of children who consumed fish, millet, lettuce, and papaya once a month or less was 91.9%, 91.7%, 89.6%, and 39.8%, respectively. (Table 3).

**Table 3:** Dietary patterns of school children, southwest Ethiopia, 2016.

Food Group	Food	Daily	3–6 days/week	1–2 days/week	1–3 day/month	<1/month or never
**Vegetables**	Abyssinian cabbage	291(37.9%)	186(24.3%)	240(31.3%)	21(2.7%)	29(3.8%)
	Cabbage	50(6.5%)	41(5.3%)	273(35.6%)	311(40.5%)	92(12.0%)
	Lettuce	3(0.4%)	8(1.0%)	37(4.8%)	32(4.2%)	687(89.6%)
	Kolard green	0	19(2.5%)	19(2.5%)	30(3.9%)	699(3.0%)
	Potato	239(31.2%)	210(27.4%)	238(31.0%)	34(4.4%)	46(6.0%)
**Root**	Taro root	367(47.8%)	99(12.9%)	156(20.3%)	84(11.0%)	61(8.0%)
**Fruits**	Banana	443(57.8%)	143(18.6%)	119(15.5%)	24(3.1%)	38(5.0%)
	Papaya	78(10.2%)	117(15.3%)	176(22.9%)	91(11.9%)	305(39.8%)
**Cereals**	Corn	407(53.1%)	68(8.9%)	52(6.8%)	22(2.9%)	218(28.4%)
	Millet	9(1.2%)	14(1.8%)	23(3.0%)	18(2.3%)	703(91.7%)
**Animal products**	Fish	0	0	16(2.1%)	46(6.0%)	705(91.9%)
	Meat	5(0.7%)	21(2.7%)	262(34.2%)	427(55.7%)	52(6.8%)
	Milk	43(5.6%)	70(9.1%)	326(42.5%)	237(30.9%)	91(11.9%)
	Egg	48(6.3%)	149(19.4%)	323(42.1%)	179(23.3%)	68(8.9%)
**Drinks**	Coffee	381(49.7%)	108(14.1%)	130(16.9%)	99(12.9%)	49(6.4%)
	Tea	630(82.1%)	70(9.1%)	46(6.0%)	3(0.4%)	18(2.3%)

### Children characteristics

The mean age of children is 115.8 (±18.9) months and more than half (52.5%) are girls. Based on clinical examination, the proportion of children with grade 1 and grade 2 goiter was 12% and 4%, respectively, making the total goiter rate (TGR) 16%. The prevalence of clinically assessed total goiter is significantly higher in girls (19.8%) than boys (11.9%) (p = 0.009) (Table 4).

**Table 4:** Child characteristics and association with goiter rate, southwest Ethiopia, 2016.

Child characteristic	Categories	Goiter rate				P value
		Grade 0 N (%)	Grade 1 N (%)	Grade 2 N (%)	Total Goiter Rate N (%)	
**Age (in months)**	72 to 95	35(81.4)	7(16.3)	1(2.3)	8(18.6)	0.484
	96 to 119	174(80.9)	31(14.4)	10(4.7)	41(19.1)	
	120 to 143	435(85.5)	54(10.6)	20(3.9)	74(14.5)	
**Gender**	Boy	324(88.0)	35(9.5%)	9(2.4%)	44(11.9)	0.009
	Girl	320(80.2)	57(14.3)	22(5.5)	79(19.8)	
**Total**		644(84.0)	92(12.0)	31(4.0)	31(16.0)	

### Urinary iodine concentration (UIC)

The median UIC values of single spot urine tests are presented in Table 5. The overall median of UIC is 74.0 μg/L (IQR 36.6–134.2 μg/L). Boys (81.6) are found to have a higher median UIC than girls (73.1) and it is marginally significant (p = 0.05). When the median UIC is disaggregated by age and district, significant difference between the age groups (p = 0.022) and district was seen. Sheko district have significantly lower median UIC level than Mizan-Aman and Debrework (p<0.001). There is no statistically significant difference in the median UIC level between groups of wealth index (p = 0.54). Based on the UIC category, the proportion of children with severe, moderate and mild iodine deficiency was 13.7%, 18.6%, and 26.5%, respectively. Whereas among the salt samples obtained from households, 428 (55.8%) have ≥15ppm of iodine content, while 35.6% have <15ppm and 8.6% have none.

**Table 5:** Urinary iodine concentration in relation to sex, age and location among school children in southwest Ethiopia, 2016.

	UIC (μg/l)			UIC category					
				Severe	Moderate	Mild	Adequate	Above req.	Excess
	N	Median	IQR	N (%)	N (%)	N (%)	N (%)	N (%)	N (%)
**Total**	767	74.0	36.6, 134.2	105(13.7)	143(18.6)	203(26.5)	194(25.3)	78(10.2)	44(5.7)
**Gender** ^a^									
Boy	368	81.6	40.4, 171.2	42(11.4)	71(19.3)	87(23.6)	94(25.5)	47(12.8)	27(7.3)
Girl	399	73.1	36.1, 120.8	63(15.8)	72(18.0)	116(29.1)	100(25.1)	31(7.8)	17(4.3)
**District** ^b^									
Mizan-Aman	248	88.9	41.9, 175.6	28(11.3)	53(21.4)	48(19.4)	67(27.0)	29(11.7)	23(9.3)
Sheko	225	68.2	24.9, 107.7	44(19.6)	44(19.6)	65(28.9)	52(23.1)	16(7.1)	4(1.8)
Debrework	294	86.0	45.0, 146.8	33(11.2)	46(15.6)	90(30.6)	75(25.5)	33(11.2)	17(5.8)
**Age** ^b^ **(months)**									
72 to 95	43	88.9	33.5, 196.4	7(16.3)	7(16.3)	9(20.9)	11(25.6)	5(11.6)	4(9.3)
96 to 119	215	93.0	41.4, 172.6	22(10.2)	38(17.7)	52(24.2)	62(28.8)	31(14.4)	10(4.7)
120 to 143	509	72.5	36.1, 123.2	76(14.9)	98(19.3)	142(27.9)	121(23.8)	42(8.3)	30(5.9)
**Wealth Index.** ^b^									
Lowest 40%	307	74.0	36.5, 126.0	39(12.7)	59(19.2)	83(27.0)	87(28.3)	22(7.2)	17(5.5)
Middle 40%	307	79.1	37.4, 164.4	39(12.7)	56(18.2)	77(25.1)	74(24.1)	40(13.0)	21(6.8)
Highest 20%	153	72.8	34.8, 116.0	27(17.6)	28(18.3)	43(28.1)	33(21.6)	16(10.5)	6(3.9)

^a^- Mann-Whitney U test,

^b^- Kruskal Wallis test

UIC = *Urinary iodine concentration*, Req. = *requirement*

### Correlation of dietary pattern and urinary iodine level

**Fig 1:**
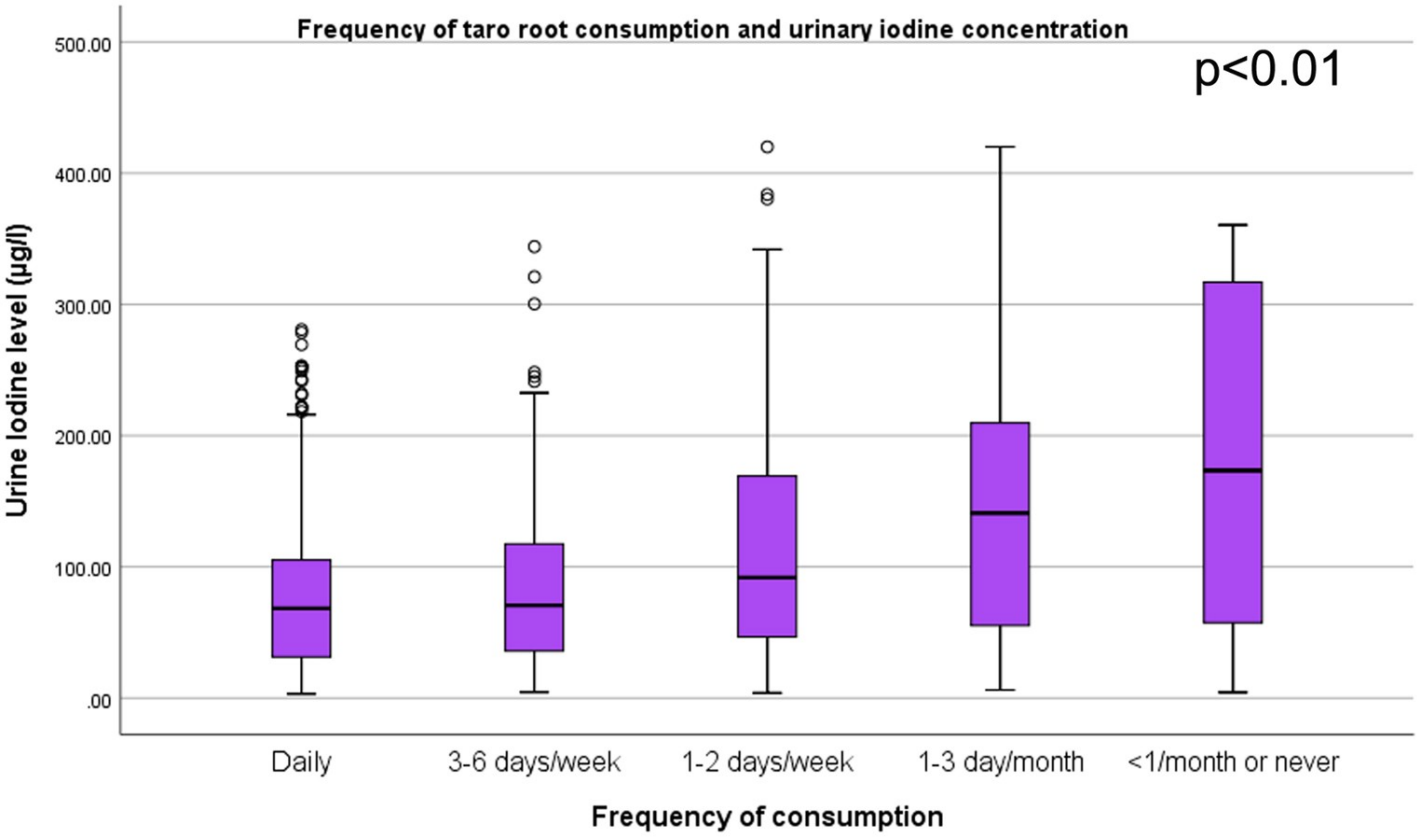
Taro root consumption and urinary iodine level among school children. Box plot diagram showing the urinary iodine excretion (UIC) level (μg/l) across the level of taro root consumption among school children in southwest Ethiopia. The box-plot shows, the UIC among children who consume taro root daily (n = 367, median = 68.4, IQR = (31.2, 105.3)), 3–6 days per week (n = 99, median = 70.6, IQR = (35.5, 117.6)), 1–2 days per week (n = 156, median = 91.8, IQR = (46.5, 172.6)), 1–3 days per month (n = 84, median = 141.1, IQR = (54.7, 210.3)), and less than 1 per month or never (n = 61, median = 173.4, IQR = (57.4, 173.4)).

**Fig 2:**
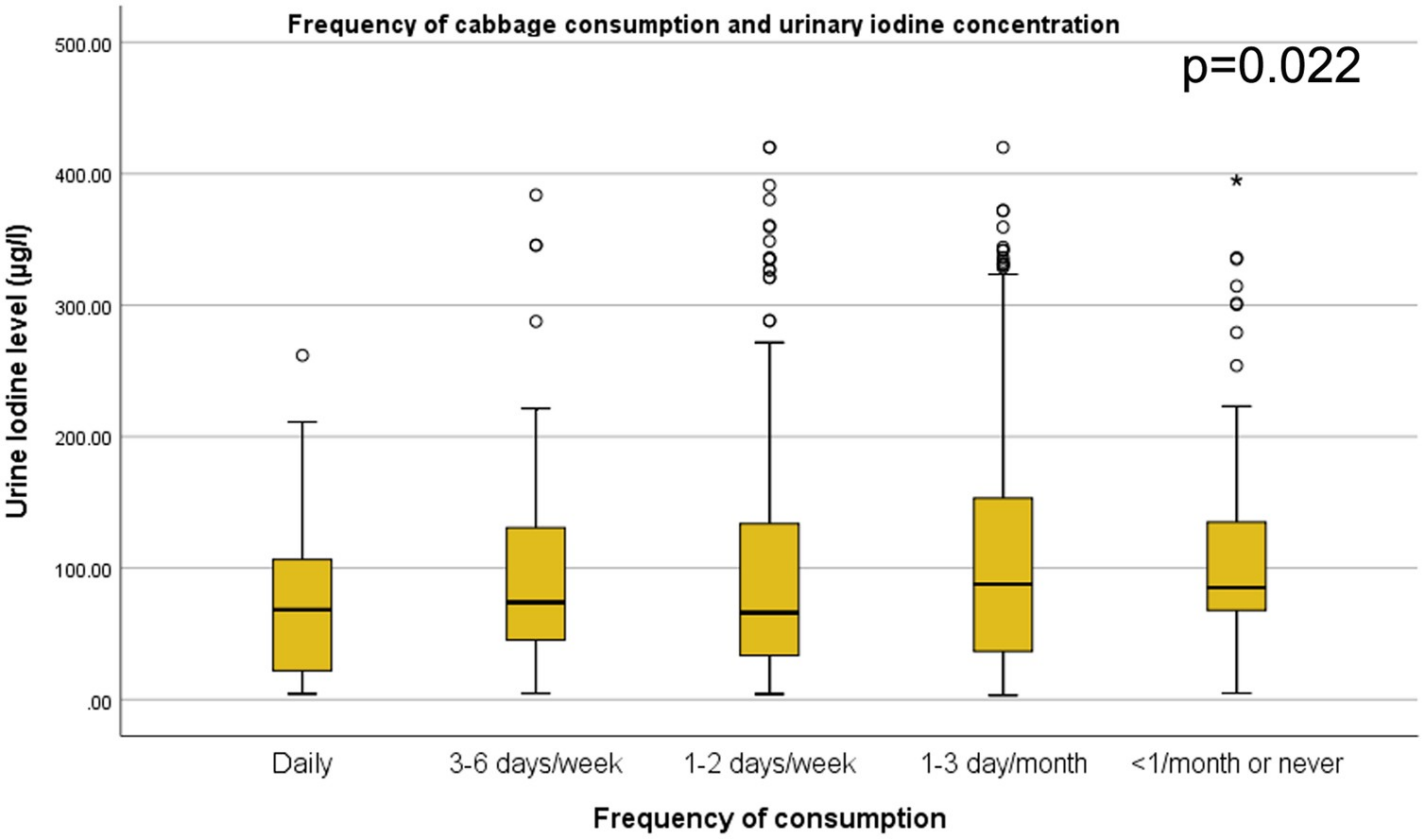
Cabbage consumption and urinary iodine level among school children. Box-plot diagram showing the urinary iodine excretion (UIC) level (μg/l) across level of cabbage consumption among school children in southwest Ethiopia. The box-plot shows, UIC among children who consume cabbage daily (n = 50, median = 68.3, IQR = (21.5, 107.4)), 3–6 days per week (n = 41, median = 74.0, IQR = (45.1, 140.5)), 1–2 days per week (n = 273, median = 66.1, IQR = (33.5, 134.9)), 1–3 days per month (n = 311, median = 87.8, IQR = (36.7, 153.9)), and less than 1 per month or never (n = 92, median = 85.1, IQR = (67.5, 135.3)).

**Fig 3:**
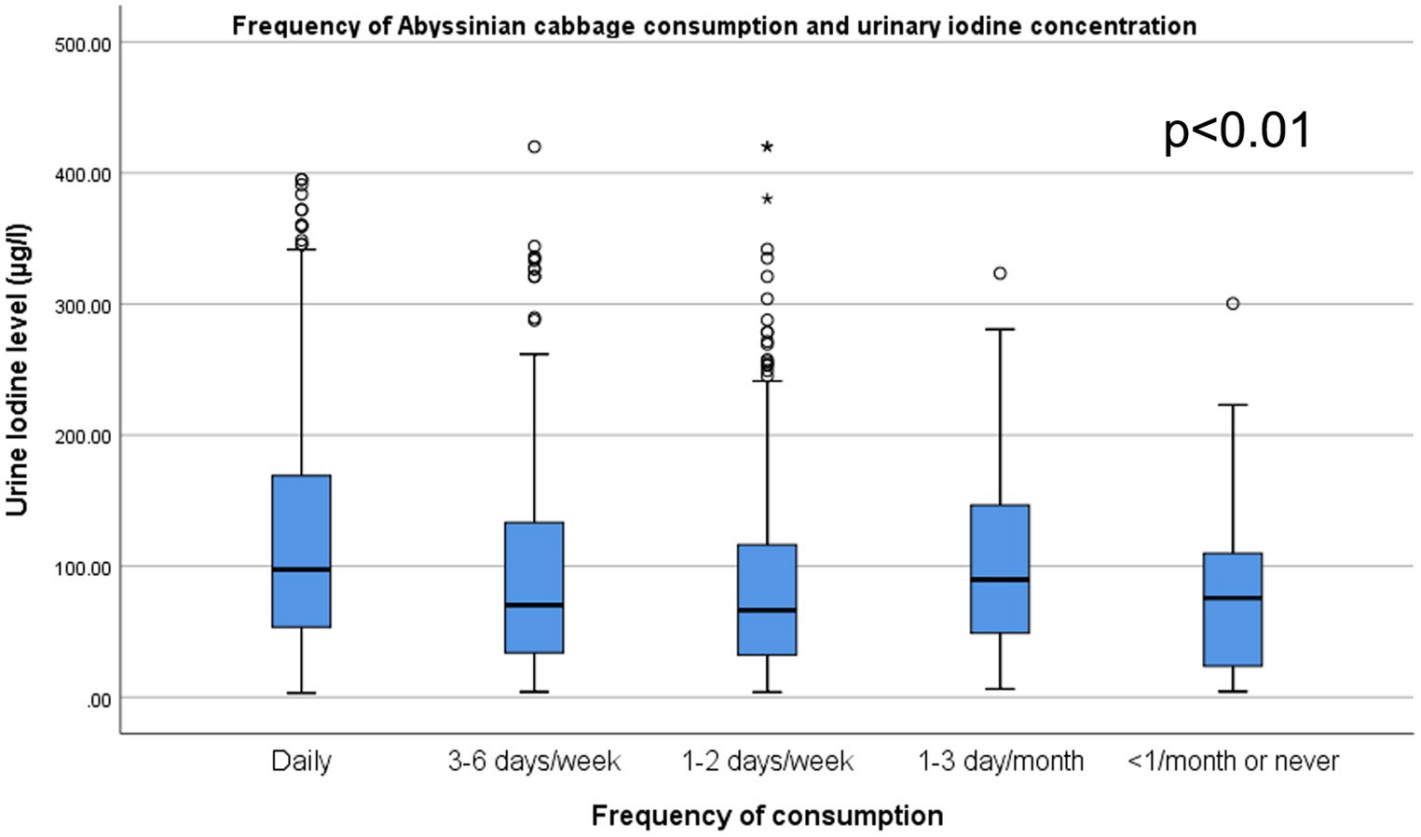
Abyssinian cabbage consumption and urinary iodine level among school children. Box-plot diagram showing the urinary iodine excretion (UIC) level (μg/l) across level of Abyssinian cabbage consumption among school children in southwest Ethiopia. The box-plot shows, UIC among children who consume Abyssinian cabbage daily (n = 291, median = 97.4, IQR = (52.0, 170.0)), 3–6 days per week (n = 186, median = 70.3, IQR = (38.9, 133.7)), 1–2 days per week (n = 240, median = 66.3, IQR = (32.0, 116.3)), 1–3 days per month (n = 21, median = 89.7, IQR = (40.8, 161.3)), and less than 1 per month or never (n = 29, median = 75.6, IQR = (23.1, 116.5)).

**Fig 4:**
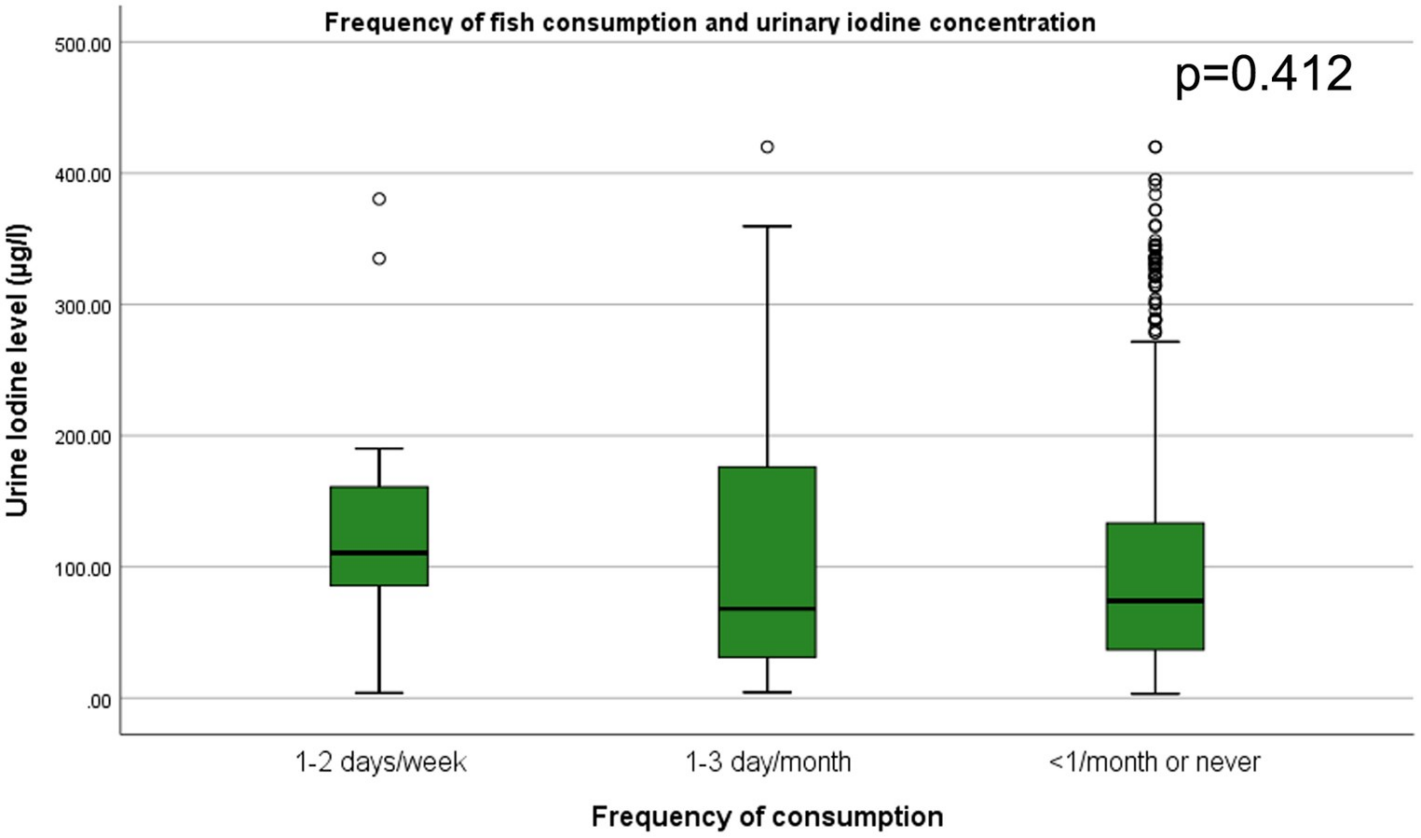
Fish consumption and urinary iodine level among school children. Box-plot diagram showing the urinary iodine excretion (UIC) level (μg/l) across level of fish consumption among school children in southwest Ethiopia. The box-plot shows, UIC among children who consume fish 1–2 days per week (n = 16, median = 110.6, IQR = (29.2, 164.4)), 1–3 days per month (n = 46, median = 67.9, IQR = (30.3, 176.3)), and less than 1 per month or never (n = 705, median = 74, IQR = (36.8, 133.3)).

Figs 1–4 shows, as the frequency of taro root and cabbage consumption at the population level decreases, the median urinary iodine excretion level increases. It is interesting to note that, as the frequency of fish and Abyssinian cabbage consumption decrease, the median urinary iodine excretion level decreases.

### Multiple linear regression analysis

To identify independent factors associated with urinary iodine concentration, we employed a multivariate linear regression model. Age and sex of children, wealth index, and frequency of consumption of fish, taro root, Abyssinian cabbage, cabbage, potato, millet, and banana were included in the model. Child age, sex, consumption of taro root, cabbage, Abyssinian cabbage, banana, and millet were significantly associated with urinary iodine level. Urinary iodine concentration (UIC) decreases as the consumption of taro root (β = -27.4, 95%CI = -22.9, -31.8), cabbage (β = -11.7, 95%CI = -5.7, -17.6), and millet (β = -10.3, 95%CI = -1.6, -19.0) increases. On the other hand, UIC increases as the consumption of Abyssinian Cabbage (β = 12.4, 95%CI = 6.7, 18.2) and banana (β = 5.6, 95%CI = 0.01, 11.2) increases. On average as the age of child is higher by a month the urinary iodine concentration is lower by 0.7ug/l (β = -0.7, 95%CI = -1.1, -0.4). Similarly, on average girls have lower urinary iodine level (β = -22.3, 95%CI = -33.8, -10.8). (Table 6).

**Table 6:** Multivariate linear regression of factors associated with urinary iodine level among school children in southwest Ethiopia, 2016.

	Coefficients		T	Sig.	95.0% Confidence Interval for B	
	B	SE			Lower Bound	Upper Bound
(Constant)	132.129	51.113	2.585	.010	31.788	232.470
Child age	-.713	.176	-4.043	.000*	-1.059	-.367
Child sex	-22.313	5.871	-3.801	.000*	-33.839	-10.788
Wealth index	-1.628	3.931	-.414	.679	-9.346	6.090
Taro root	-27.373	2.257	12.130	.000*	-31.803	-22.943
Cabbage	-11.659	3.036	3.840	.000*	-17.619	-5.698
Fish	4.282	8.134	-.526	.599	-11.686	20.250
Abyssinian Cabbage	12.447	2.932	-4.245	.000*	6.691	18.202
Millet	-10.256	4.432	2.314	.021*	-18.957	-1.555
Banana	5.607	2.853	-1.965	.050	.007	11.207
Potato	1.353	2.839	.477	.634	-4.221	6.928

*- statistically significant at α = 0.05

## Discussion

This study assessed the magnitude of iodine deficiency using total goiter rate and urinary iodine excretion level in southwest Ethiopia and its correlates with dietary intake and socio-demographic determinants. We selected children from school since the primary school enrollment rate is above 90% for both sexes in Ethiopia [32], we assumed that it is representative of all school-age children. Based on the clinical examination of the thyroid gland, 12% had a palpable goiter and 4% had a visible goiter with an overall TGR of 16%. This result is lower as compared to studies done in northeast Ethiopia, which reported a TGR of 37.6% and 62.1% [33, 34], and 35.2% from south Ethiopia [35]. The main reason for this discrepancy could be attributed to their agro-ecology variation, in which, most of the aforementioned findings are from the highlands in contradiction of the present study which is relatively low land. Nevertheless, the magnitude is unacceptably high and it is a major public health problem as the prevalence exceeds the WHO threshold criteria of TGR above 5%. Based on the WHO recommendation, a TGR of 5% and above in school children aged 6 to 12 years of age is considered as a sign of the presence of a major public health problem.

In this study, girls (19.8%) have significantly higher total goiter rate than boys (11.9%), which is coherent with previous studies including the systematic reviews [33, 34, 36]. Laboratory results also indicate girls have a lower median UIC than boys and it is statistically marginally significant (p = 0.05). Such differences are expected as the androgen hormones in males are stimulatory while estrogen hormone in females has an inhibitory effect on thyroid growth [37]. On the other hand, the prevalence of iodine deficiency based on UIC is 58.8%, which is higher than the TGR and is almost three folds and suggests that most of the participants have a sub-clinical or asymptomatic iodine deficiency. Our finding is concordant with the findings reported by Hailu and his colleagues which reported 57% of children to have low urinary iodine level [29]. This implies that if no measure is taken, it is likely that the sub-clinical state will consequently end in endemic goiter and other related disorders.

Dietary intake of children was estimated using modified Hellen Keller’s food frequency questionnaire. Accordingly, higher consumption of Abyssinian cabbage, taro root, banana, corn, and potato were observed. Almost half (47.8%) and 53.1% of children consume taro root and corn, respectively, on daily bases, these foods are the staple diet to the study area and are postulated to be goitrogenic food. Taro root is a common staple food that grows in the study area unlike in the north and central Ethiopia, where they do not grow and consume it. Fish and lettuce consumption are also rare and such dietary practice may contribute to iodine deficiency. On average those children who consume fish, Abyssinian cabbage, and banana more frequently have higher urinary iodine level. In contrast, those who consume taro root and millet more frequently have lower urinary iodine level.

In the multivariate linear regression model, age and sex of the child, consumption of taro root, millet, cabbage, Abyssinian cabbage, and banana were the significant factors associated with urinary iodine level.

On average higher age groups have a lower urinary iodine level, which is supported by several studies [29, 34, 38]. This could be explained by the increase in demand for iodine related to puberty. Girls have significantly lower urinary iodine level than boys, which is consistent with several studies [29, 34, 35]. Urinary iodine linearly decreases as the frequency of consumption of taro, cabbage, and millet increases. Taro root is known in its high content of phytate, a compound which inhibits iodine absorption [39]. Similarly, cabbage and millet are also associated with suppression of thyroid hormones that might contribute to the lower urinary iodine level [40, 41]. The previous study in south Ethiopia identified frequent consumption of cassava as a significant predictor of goiter [42], which has a similar chemical composition with taro root. In the contrary, those who consume Abyssinian cabbage and banana more frequently, have higher urinary iodine level on average. The iodine content of banana and its contribution is supported by a study done on the biochemical analysis of foods [43]. However, there is a dearth of literature on nutritional value and biochemical content of Abyssinian cabbage, which calls for further research. In the multivariate analysis, the association between consumption of fish and iodine deficiency is not statistically significant. The extremely lower consumption of fish in the study participants could make the comparison unbalanced and lead to non-significant association. Moreover, the fish supply for the study area is from Awasa and Arba-minch, which are known in their low iodine content of the fish [44].

The use of urinary iodine level along with goiter grading is an important strength of the present study. This gives an opportunity to consider an asymptomatic iodine deficiency apart from goiter. Moreover, this study assessed the association of urinary iodine with food items that are available in that specific area. Furthermore, we used a larger sample size that increases the power of the study.

The findings of this study need to be interpreted considering the following limitations. First, we measured the dietary pattern based on the frequency of feeding and food types only. We did not estimate the amount and type of food items exactly taken by the child. We assessed the estimated frequency for each food type retrospectively. As a result, we could not estimate the exact amount of increase/decrease of UIC per number of servings or grams of the food taken by the child. Second, there might be an overestimation of food types that the community considers as good, leading to social-desirability bias. Nevertheless, this might not affect the association between dietary pattern and urinary iodine level as the overestimation is non-differential. Third, we did not collect and control for the hygienic practices of the mother/caregiver that might have an association with iodine deficiency. At last, we did not assess the level of other micronutrients, which might affect iodine absorption. Hence, part of the association could be attributable to the coexistence of other micronutrient deficiencies rather than dietary pattern. However, as the distribution of co-existing micronutrient deficiencies could be random for those with and without iodine deficiency, the effect measure might not be biased.

## Conclusions

Iodine deficiency is still an important public health problem in southwest Ethiopia. Both over-consumption of goitrogenic foods and under-consumption of iodine-rich foods were prevalent and associated with lower urinary iodine level. Frequent consumption of Abyssinian cabbage was observed and associated with higher urinary iodine level. We recommend, dietary counseling services in addition to universal salt iodization to lower the problem of iodine deficiency. Moreover, further research employing robust research designs with better estimation of the amount of food taken and more biochemical analysis of foods consumed, especially Abyssinian cabbage, is recommended to answer the causal relationship between dietary pattern and iodine deficiency.

## Supporting information

S1 File English version questionnaire. (PDF)

S2 File Amharic version questionnaire. (PDF)

S1 Dataset The minimal dataset of the study. (CSV)
