# Supplementary material for: Dietary pattern and its association with iodine deficiency among school children in southwest Ethiopia; A cross-sectional study
Source: PLoS One. 2019 Aug 13;14(8):e0221106. doi: 10.1371/journal.pone.0221106 (PMC6692009; doi:10.1371/journal.pone.0221106)
Supplement: S1 File — (PDF) [file pone.0221106.s001.pdf]

English version Questionnaire

Questionnaire code: __________
Woreda name: ____________ Woreda code: ____________ Altitude: _________
School name: ______________ School code: ______________
Date: _______________

Part I - socio-demographic characteristics

S.No	Questions	Responses
101	What is your age?	__________ Years
102	What is your religion?	1. Orthodox<br>2. Muslim<br>3. Protestant<br>4. Other ____________________
103	What is your ethnicity?	1. Bench<br>2. Amhara<br>3. Sheko<br>4. Keffa<br>5. Tigre<br>6. Other ____________________
104	Place of residence	1. Urban<br>2. Rural
105	What is your marital status	1. Single<br>2. Married<br>3. Divorced<br>4. Widowed<br>5. Separated
106	What is the highest level of school you attended?	1. Cannot read and write<br>2. Primary education(1-8)<br>3. High school education(9-12)<br>4. Tertiary education(college, university)
107	What is the highest level of school your husband/partner attended?	1. Cannot read and write<br>2. Primary education(1-8)<br>3. High school (9-12)<br>4. Tertiary education(college, university)
108	What is your occupation?	1. Housewife<br>2. Farmer<br>3. Merchant<br>4. Government employee<br>5. Self-employee<br>6. Daily laborer<br>7. Other________________
109	What is your husband/partner occupation?	1. Farmer<br>2. Merchant<br>3. Government employee<br>4. Self-employee

		5. Daily laborer<br>6. Other_________________
110	Family size (number)	______________
111	Estimated family monthly income	_____________ Ethiopian Birr

Part II - Household Wealth Assessment

S.no	Questions	Responses
201	Main material of the floor of house	1. Soil/sand<br>2. Wood planks<br>3. Cement<br>4. Ceramic tiles<br>5. Others(specify) _______________
202	Main material of roof	1. Thatch<br>2. Corrugated iron/metal<br>3. Cement<br>4. Others (specify) ________________
203	Does member of this household own,	
	A bicycle?	1. Yes 2. No
	A motorcycle?	1. Yes 2. No
	An animal-drawn cart?	1. Yes 2. No
	A car or truck?	1. Yes 2. No
204	Does household own any agricultural land	1. Yes ___________<br>2. No
205	Does this household own any livestock, herds, other farm animals, or poultry?	1. Yes<br>2. No
206	Does the household own the following?<br>If yes please specify the amount	
	1. Cows/bulls/oxen	1. Yes________ 2. No
	2. Horses/donkeys/mules	1. Yes________ 2. No
	3. Goats	1. Yes________ 2. No
	4. Sheep	1. Yes________ 2. No
	5. Chickens	1. Yes________ 2. No
	6. Beehives	1. Yes________ 2. No
207	How many of the following does this household own?	1. Teff (kuntal)___________<br>2. Coffee (kilo)____<br>3. Gold (gram) _____________<br>4. Silver (gram) _____________<br>5. Spices (kilo)_____________
208	Does this household have a bank or microfinance saving account?	1. Yes 2. No
209	Does your household have:	
	Electricity?	1. Yes 2. No
	A watch/clock?	1. Yes 2. No
	A radio?	1. Yes 2. No
	A television?	1. Yes 2. No
	A mobile telephone?	1. Yes 2. No
	A non-mobile telephone?	1. Yes 2. No

	A refrigerator?	1. Yes 2. No
	A table? A chair?	1. Yes 2. No
	A bed with cotton/sponge/spring mattress?	1. Yes 2. No
	An electric oven?	1. Yes 2. No
	A kerosene lamp/pressure lamp?	1. Yes 2. No

<u>Part III – Iodized salt related awareness of the mother/caregiver and source of water</u>

S.No.	Questions	Response categories
301	Have you ever heard about iodized salt?	1. Yes<br>2. No
302	If yes to Q 301, where do you get the information?	1. Health workers<br>2. Friends<br>3. Relative<br>4. Family<br>5. Advertisements<br>6. Others (specify)____________
303	Could you able to differentiate iodized salt from non-iodized salt?	1. Yes<br>2. No
304	If yes to Q 303, how do you differentiate iodized salt?	1. See the labeling<br>2. By the taste<br>3. By color<br>4. Others (specify)_______
304	Do you use salt for food?	1. Yes<br>2. No
305	If yes to Q 304, when do you add salt to the food	1. Initial<br>2. Mid-point<br>3. At the end<br>4. After completed
306	What is the main source of drinking water for members of your household?	<u>Piped water/supply water</u><br>Piped inside dwelling .... 11<br>Piped to yard/plot........................ 12<br>Public tap.................................... 13<br><u>Water from spring</u><br>Protected well/spring .... 21<br>Unprotected well/spring............... 22<br><u>Water from Dug well</u><br>Protected well .... 31<br>Unprotected well .... 32<br><u>Water form borehole</u><br>Borehole in yard/plot................... 41<br>Public borehole .... 42<br><u>Surface water</u><br>Pond/lake/River/stream/spring/Dam……….51<br><u>Rain water</u> .... 61<br><u>Tanker truck</u>................................... 71<br><u>Vendor</u>.. 81

		<u>Bottled water</u>.................................. 91<br>No fixed facility................................ 96<br>Other (specify) 99
307	What is the main source of water used by your household for other purposes such as cooking and hand washing?	<u>Piped water/supply water</u><br>Piped inside dwelling .... 11<br>Piped to yard/plot........................ 12<br>Public tap.................................... 13<br><u>Water from spring</u><br>Protected well/spring .... 21<br>Unprotected well/spring............... 22<br><u>Water from Dug well</u><br>Protected well .... 31<br>Unprotected well .... 32<br><u>Water form borehole</u><br>Borehole in yard/plot................... 41<br>Public borehole .... 42<br><u>Surface water</u><br>Pond/lake/River/stream/spring/Dam……….51<br><u>Rain water</u> .... 61<br><u>Tanker truck</u>.................................. 71<br><u>Vendor</u>.. 81<br><u>Bottled water</u>.................................. 91<br>No fixed facility................................ 96<br>Other (specify) 99

Part IV - Helen Keller Modified food frequency questionnaire (FFQ)

Food frequency questionnaire to be answered by mothers					
Food item	≥ 1x/day	3-6x/ week	1-2x/w	≤ 2x/month	Never
**Cereals/cereal based food items**					
Teff					
Sorghum					
Millet					
Corn					
Wheat					
Barley					
Macaroni					
Spaghetti					
Rice					
**Vegetables**					
Tomato					
Potatoes					
Beetroot					
Collard green					
Cabbage					
Abyssinian cabbage					
Carrot					
Lettuce					
Sweet potatoes					
Cocumber					
**Roots**					
Godere					
Ensete					
**Pulses**					
Beans					
Peas					
Kidney beans					
Lentils					
Chickpeas					
Haricot beans					
**Fruits**					
Orange					
Banana					
Mango					
Avocado					
Papaya					
Guava					

Animal products					
Egg					
Beef					
Milk					
Fish					
Chicken					

Part V - Child characteristics

Sn. No	Questionnaire	Responses
501	Child ID	____________
502	What is the age of a child?	_____year and _____months
503	What is the sex of a child?	1. Boy<br>2. Girl
504	School grade of the child?	1. Grade 1<br>2. Grade 2<br>3. Grade 3<br>4. Grade 4<br>5. Grade 5<br>6. Grade 6

Part VI - Anthropometric and thyroid palpation of the child

Child *ID* ________________ Age_____________

S.No.	Activity	Result	remark
601	Height	____________cm	
602	Weight	____________Kg	
603	Thyroid gland palpation and staging	1. grade o<br>2. grade 1<br>3. grade 2	

Part VII - salt sample test reporting format

Child *ID* _______________ Age____________

S.No.	Type of test	Result	Remark
701	Simple rapid test	1. 0 ppm (white)<br>2. <15ppm (light grey)<br>3. >15ppm (dark grey)	

Part VIII - Laboratory result reporting format

Name of health institution-------------

Child *ID* -----------

Age……………

	Type of test	Result	Remark
1	Urinary iodine concentration	UIC__________μg/L	

Name of laboratory investigator--

Signature-------------------------

Date------------------------------
