# Supplementary material for: Dietary pattern and its association with iodine deficiency among school children in southwest Ethiopia; A cross-sectional study
Source: PLoS One. 2019 Aug 13;14(8):e0221106. doi: 10.1371/journal.pone.0221106 (PMC6692009; doi:10.1371/journal.pone.0221106)
Supplement: S2 File — (PDF) [file pone.0221106.s002.pdf]

# መጠይቅ

የመጠይቅ ኮድ: __________

የወረዳ ስም: ________ የወረዳ ኮድ: ________ ከፍታ: ______
የትምህርት ቤት ስም: _________ የት / ቤት ኮድ: _________
የመጠይቅ ቀን: ________________

## ክፍል 1 - ማህበራዊ ና ስነ-ሕዝብ መረጃ

| ተ ቁ | ጥያቄዎች | ምላሾች |
|---|---|---|
| 101 | አሁን ዕድሜዎ ስንት ነው? | ______ ዓመታት |
| 102 | ሃይማኖትዎ ምንድን ነው? | 1. ኦርቶዶክስ<br>2. ሙስሊም<br>3. ፕሮቴስታንት<br>4. ሌላ (ይጥቀሱ) ____________ |
| 103 | የእርስዎ ብሄር ምንድነው? | 1. ቤንች<br>2. አማራ<br>3. ሸኮ<br>4. ከፋ<br>5. ትግሬ<br>6. ሌላ (ይጥቀሱ)____________ |
| 104 | የመኖሪያ ቦታ | 1. ከተማ<br>2. ገጠር |
| 105 | የጋብቻ ሁኔታዎ ምንድነው? | 1. ያላገባ<br>2. ያገባ<br>3. የተፋታ<br>4. የሞተባት<br>5. የተለያዩ |
| 106 | እርስዎ የተማሩበት ከፍተኛው ትምህርት ቤት ደረጃ ስንት ነው? | 1. ማንበብና መጻፍ አይቻልም<br>2. የመጀመሪያ ደረጃ ትምህርት (1-8)<br>3. የሁለተኛ ደረጃ ትምህርት (9-12)<br>4. ከፍተኛ ደረጃ ትምህርት (ኮሌጅ, ዩኒቨርሲቲ) |
| 107 | የባለቤትዎ / የትዳር ጓደኛዎ ከፍተኛ ትምህርት ደረጃ ስንት ነው? | 1. ማንበብና መጻፍ አይቻልም<br>2. የመጀመሪያ ደረጃ ትምህርት (1-8)<br>3. የሁለተኛ ደረጃ ትምህርት (9-12)<br>4. ከፍተኛ ደረጃ ትምህርት (ኮሌጅ, ዩኒቨርሲቲ) |
| 108 | ሥራዎ ምንድን ነው? | 1. የቤት እመቤት<br>2. ገበሬ<br>3. ነጋዴ<br>4. የመንግስት ሰራተኛ<br>5. ለራስ-ሰራተኛ<br>6. የቀን ሰራተኛ<br>7. ሌላ (ይጥቀሱ)__________ |
| 109 | የባለቤትዎ / የትዳር ጓደኛዎ ሥራ ምንድን ነው?? | 1. ገበሬ<br>2. ነጋዴ<br>3. የመንግስት ሰራተኛ<br>4. ለራስ-ሰራተኛ<br>5. የቀን ሰራተኛ<br>6. ሌላ (ይጥቀሱ)__________ |

| | | |
|---|---|---|
| 110 | የቤተሰብ ብዛት (ቁጥር) | _________ |
| 111 | ግምታዊ የቤተሰብ ወርሃዊ ገቢ | ________ የኢትዮጵያ ብር |

## ክፍል 2 - የቤተሰብ ሀብት ግምገማ

| ተ ቁ | ጥያቄዎች | ምላሾች |
|---|---|---|
| 201 | በቤት ውስጥ ወለሉ የተሰራው ቁሳቁስ ምንድን ነው? | 1. አፈር / አሸዋ<br>2. የእንጨት ሰሌዳዎች<br>3. ሲሚንቶ<br>4. ሴራሚክ ሰድሎች<br>5. ሌሎች (ዝርዝር ይግለጹ)_________ |
| 202 | በቤት ውስጥ ጣራውየተሰራው ቁሳቁስ ምንድን ነው? | 1. 1. ሣር<br>2. 2. የተጣራ ብረት / ብረት<br>3. 3. ሲሚንቶ<br>4. 4. ሌሎች (ይግለጹ) __________ |
| 203 | የዚህ ቤት አባል የሚከተለው የሚከተሉትን በባለቤትነት ይይዛል? | |
| | ብስክሌት? | 1. አዎ 2. አይደለም |
| | ሞተርሳይክል? | 1. አዎ 2. አይደለም |
| | እንስሳ-መርሻ ጋሪ? | 1. አዎ 2. አይደለም |
| | የቤት መኪና ወይም የጭነት መኪና? | 1. አዎ 2. አይደለም |
| 204 | የእርስዎ ቤተሰብ ማንኛውም የእርሻ መሬት ይኖረዋልን?<br>አዎ ከሆነ እባክዎን መጠኑን ይግለጹ | 1. አዎ _______<br>2. አይደለም |
| 205 | ይህ ቤተሰብ ከብቶች, ከብቶች, ሌሎች የእርሻ እንስሳት ወይም የዶሮ እርባታ አለው? | 1. አዎ<br>2. አይደለም |
| 206 | ይህ ቤተሰብ የሚከተሉትን በባለቤትነት ይይዛል?<br>አዎ ከሆነ እባክዎን መጠኑን ይግለጹ | |
| | 1. ላሞች / በሬዎች / በሬዎች | 1. አዎ _________2. አይደለም |
| | 2. ፈረሶች / አህዮች / ሞለሶች | 1. አዎ _________2. አይደለም |
| | 3. ፍየሎች | 1. አዎ _________2. አይደለም |
| | 4. የበጎች | 1. አዎ _________2. አይደለም |
| | 5. ዶሮዎች | 1. አዎ _________2. አይደለም |
| | 6. የንብ ቀፎዎች | 1. አዎ _________2. አይደለም |
| 207 | ይህ ቤተሰብ የሚከተሉትን በባለቤትነት ይይዛል?<br>አዎ ከሆነ እባክዎን መጠኑን ይግለጹ | |
| | 1. ጤፍ (ኩንታል) | 1. አዎ _________2. አይደለም |
| | 2. ቡና (ኪሎ) | 1. አዎ _________2. አይደለም |
| | 3. ወርቅ (ግራም) | 1. አዎ _________2. አይደለም |
| | 4. ብር (ግራም) | 1. አዎ _________2. አይደለም |
| | 5. ቅመሞች (ኪሎ) | 1. አዎ _________2. አይደለም |
| 208 | ይህ ቤተሰብ ባንክ ወይም ማይክሮ ፋይናንስ ቁጠባ ሂሳብ አለው? | 1. አዎ 2. አይደለም |
| 209 | የእርስዎ ቤተሰብ እነኚህ አለው? | |
| | የኤሌክትሪክ ኃይል? | 1. አዎ 2. አይደለም |
| | ሰዓት / የግድግዳ ሰዓት? | 1. አዎ 2. አይደለም |
| | ሬዲዮ? | 1. አዎ 2. አይደለም |
| | ቴሌቪዥን? | 1. አዎ 2. አይደለም |

| | | |
|---|---|---|
| | የሞባይል ስልክ? | 1. አዎ 2. አይደለም |
| | የሞባይል ስልክ ነው? | 1. አዎ 2. አይደለም |
| | ማቀዝቀዣ? | 1. አዎ 2. አይደለም |
| | ጠረጴዛ? ወንበር? | 1. አዎ 2. አይደለም |
| | ከጥጥ / ስፖንጅ / የጸሐይ ማቀፊያ ጥፍር? | 1. አዎ 2. አይደለም |
| | የኤሌክትሪክ ምጣድ? | 1. አዎ 2. አይደለም |
| | የጋዝ ሙቀት አምፖል | 1. አዎ 2. አይደለም |

## ክፍል 3 - ስለ ስለእናት / የተንከባካቢ አዮዲን ጨው ዕውቀት መለኪያ እና የውሃ ምንጭ

| ተ ቁ | ጥያቄዎች | ምላሾች |
|---|---|---|
| 301 | ስለ አዮዲን ጨው ሰምተው ያዉቃሉ? | 1. አዎ<br>2. አይደለም |
| 302 | ለጥያቄ ቁጥር 301 አዎን ከሆነ, መረጃውን የሚያገኙት ከየት ነው? | 1. የጤና ሰራተኞች<br>2. ጓደኞች<br>3. አንጻራዊ<br>4. ቤተሰብ<br>5. ማስታወቂያዎች<br>6. ሌሎች (ይግለጹ)________ |
| 303 | የአዮዲን ጨው አዮዲድድ ጨው መለየት ይችላሉ? | 1. አዎ<br>2. አይደለም |
| 304 | ለጥያቄ ቁጥር 303 አዎን ከሆነ, የአዮዲን ጨው እንዴት ይለያል? | 1. መለያን በመመልከት<br>2. በመጣስ<br>3. በቀለም<br>4. ሌሎች (ይግለጹ) _____ |
| 304 | ለምግብ ጨው ይጠቀማሉ? | 1. አዎ<br>2. አይደለም |
| 305 | ለጥያቄ ቁጥር 304 አዎን ከሆነ, , መቼ ጨው ይጨመርባታል? | 1. መጀመሪያ<br>2. መካከለኛ ጊዜ<br>3. መጨረሻ ላይ<br>4. ከተጠናቀቁ በኋላ |
| 306 | ለቤተሰብዎ አባላት ዋና የመጠጥ ውኃ ምንጭ ምንድነው? | የቧንቧ ውሃ<br>የቤት ውስጥ ቧንቧዎች.......................................11<br>በጓሮ ውስጥ / ፐላንት ውስጥ ቧንቧዎች ........12<br>የሕዝብ ውሃ ......................................................13<br>የምንጭ ዉሃ<br>በደንብ የተጠበቀ ..............................................21<br>በደንብ ያልተጠበቀ..............................................22<br>ከጉድጓድ ውሃ<br>በደንብ የተጠበቀ ..............................................31<br>በደንብ ያልተጠበቀ..............................................32<br>የጥልቅ ጉድጓድ ውኃ<br>የጥልቅ ጉድጓድ በጓሮ ውስጥ ............................41<br>የሕዝብ የጥልቅ ጉድጓድ ...................................42<br>የምድር ውሃ |

| | | |
|---|---|---|
| | | ኩሬ/ ሐይቅ / ወንዝ / ዥረት / ግድብ ..........51<br>የዝናብ ውሃ ........................................61<br>የታንከሪ ውሃ........................................71<br>ሻጭ ........................................81<br>የታሸገ ውሃ ........................................91<br>ምንም ቋሚ የለም ........................................96<br>ሌላ (ይጥቀሱ)........................................99 |
| 307 | በቤተሰብዎ ውስጥ እንደ ምግብ ማብሰል እና እጅን መታጠብ የመሳሰሉ ሌሎች ተግባራት የሚያከናውኑበት የውሃ ምንጭ ምንድነው? | የቧንቧ ውሃ<br>የቤት ውስጥ ቧንቧዎች........................................11<br>በጓሮ ውስጥ / ፕላንት ውስጥ ቧንቧዎች ........12<br>የሕዝብ ውሃ ........................................13<br>የምንጭ ዉሃ<br>በደንብ የተጠበቀ ........................................21<br>በደንብ ያልተጠበቀ........................................22<br>ከጉድጓድ ውሃ<br>በደንብ የተጠበቀ ........................................31<br>በደንብ ያልተጠበቀ........................................32<br>የጥልቅ ጉድጓድ ውኃ<br>የጥልቅ ጉድጓድ በጓሮ ውስጥ ............................41<br>የሕዝብ የጥልቅ ጉድጓድ ..................................42<br>የምድር ውሃ<br>ኩሬ/ ሐይቅ / ወንዝ / ዥረት / ግድብ ..........51<br>የዝናብ ውሃ ........................................61<br>የታንከሪ ውሃ........................................71<br>ሻጭ ........................................81<br>የታሸገ ውሃ ........................................91<br>ምንም ቋሚ የለም ........................................96<br>ሌላ (ይጥቀሱ)........................................99 |

## ክፍል 4 - ሔለን ካለር የተሻሻለው የምግብ መጠይቅ (FFQ)

| ለእናቶች ምላሽ እንዲሰጡ የተዘጋጁ ምግብ መጠይቅ | | | | | |
|---|---|---|---|---|---|
| የምግብ ዓይነት | ≥ 1x/ ቀን | 3-6x/ ሳምንት | 1-2x/ሳምንት | ≤ 2x/ወር | በጭራሽ |
| **ሰብሎች / በእህል ላይ የተመሰረቱ ምግቦች** | | | | | |
| ጤፍ | | | | | |
| ማሾል | | | | | |
| ዳጉሳ | | | | | |
| በቆሎ | | | | | |
| ስንዴ | | | | | |
| ገብስ | | | | | |
| ማካሮኒ | | | | | |
| ፓስታ | | | | | |
| ሩዝ | | | | | |
| አትክልቶች | | | | | |
| ቲማቲም | | | | | |
| ድንች | | | | | |
| ባፕቶት | | | | | |
| ቆስጣ | | | | | |
| ጥቅል ጎመን | | | | | |
| ሀበሻ ጎመን | | | | | |
| ካሮት | | | | | |
| ሰላጣ | | | | | |
| ስኳር ድንች | | | | | |
| ዱከር | | | | | |
| ስሮች | | | | | |
| ጎደሬ | | | | | |
| እንሰት | | | | | |
| ጥራጥሬዎች | | | | | |
| ባቄላ | | | | | |
| አተር | | | | | |

| | | | | | |
|---|---|---|---|---|---|
| የኒዶን ባቄላ | | | | | |
| ምስር | | | | | |
| አኩሪ አተር | | | | | |
| የሃሮሳ ፍሬዎች | | | | | |
| ፍራፍሬዎች | | | | | |
| ብርቱካን | | | | | |
| ሙዝ | | | | | |
| ማንጎ | | | | | |
| አቮካዶ | | | | | |
| ፓፓያ | | | | | |
| ዘይቱና | | | | | |
| የእንስሳት ምርቶች | | | | | |
| እንቁላል | | | | | |
| የበሬ ስጋ | | | | | |
| ወተት | | | | | |
| አሳ | | | | | |
| ዶሮ | | | | | |

## ክፍል 5 - የልጆች መረጃ

| ተ ቁ | ጥያቄዎች | ምላሾች |
|---|---|---|
| 501 | የልጅ መለያ ቁጥር | _________ |
| 502 | የልጁ ዕድሜ ስንት ነው? | ____ አመት እና ____ ወር |
| 503 | የልጁ ጾታ ምንድነው? | 1. ወንድ<br>2. ሴት |
| 504 | የልጁ የት / ቤት ክፍል? | 1. ክፍል 1<br>2. ክፍል 2<br>3. ክፍል 3<br>4. ክፍል 4<br>5. ክፍል 5<br>6. ክፍል 6 |

## ክፍል 6 - የልጁ አንትሮሜትሪክ እና ታይሮይድ ዕጢ

የልጅ መለያ ቁጥር___________ ዕድሜ _________

| ተ ቁ | መለኪያዎች | ውጤቶች | አስተያየት |
|---|---|---|---|
| 601 | ቁመት | _________ ሴ.ሜ | |
| 602 | ክብደት | _________ ኪ.ግ | |
| 603 | የታይሮይድ ዕጢ ምርመራ እና ደረጃ | 1. ደረጃ 0<br>2. ደረጃ 1<br>3. ደረጃ 2 | |

## ክፍል 7 - የጨው ናሙና ሪፖርት ፎርማት

የልጅ መለያ ቁጥር___________ ዕድሜ _________

| ተ ቁ | የሙከራ አይነት | ውጤቶች | አስተያየት |
|---|---|---|---|
| 701 | Simple rapid test | 1. 0 ppm (ነጭ)<br>2. <15ppm (ፈዛዛ ግራጫ)<br>3. >15ppm (ጥቁር ግራጫ) | |

## ክፍል 8 - የላቦራቶሪ ውጤት ሪፖርት ፎርማት

የጤና ተቋሙ ስም -------------

የልጅ መለያ ቁጥር-----------

ዕድሜ ...............

| ተ ቁ | የሙከራ አይነት | ውጤቶች | አስተያየት |
|---|---|---|---|
| 1 | Urinary iodine concentration<br><br>(የሽንት የ አዮዲን መጠን) | UIC________µg/L | |

የላብራቶሪ መርማሪ ስም --------------------------

ፊርማ --------------------------

ቀን ------------------------------
